# Barriers and facilitators to accessing healthcare services among elderly people living in a rural Amazonian community, Brazil

**DOI:** 10.1186/s12913-025-12945-w

**Published:** 2025-07-02

**Authors:** Gleica Soyan Barbosa Alves, Fernando José Herkrath, Rosana Cristina Pereira Parente, Rita Dariene da Silva Pinheiro, Mario Vianna Vettore

**Affiliations:** 1 Laboratório de Situação de Saúde e Gestão do Cuidado de Populações Indígenas e outros grupos vulneráveis– SAGESPI, Fundação Oswaldo Cruz, Instituto Leônidas e Maria Deane, Teresina St. 476 Adrianópolis, Manaus, Amazonas Brazil; 2 Universidade Federal do Amazonas, Instituto de Ciências Exatas e Tecnologia, Nossa Sra. do Rosário St. 3863 Tiradentes, Itacoatiara, Amazonas Brazil; 3 Escola Superior de Ciências da Saúde, Universidade do Estado do Amazonas, Carvalho Leal Ave. 1777 Cachoeirinha, Manaus, Amazonas Brazil; 4 Department of Dentistry and Oral Health, Aarhus University, Vennelyst Blvd., Aarhus C, 9 8000 Denmark

**Keywords:** Aged, Epidemiological models, Latent class analysis, Rural health services, Rural population

## Abstract

**Background:**

Health care utilisation is determined by complex interactions. Older adults living in rural areas appear to face greater barriers to health care, particularly in areas of the Amazon region. The aim of this study was to examine the relationships between predisposing, enabling, and need characteristics, and utilisation of medical services among elderly people living in rural areas in Amazon region, Brazil.

**Methods:**

A household-based cross-sectional study with 285 elderly people aged 60 years or older living in a rural community in the Amazonas state, Brazil, was conducted. Interviews were used to collect data on predisposing (demographics), enabling (socioeconomic and health services factors), and need characteristics (self-reported health measures). The outcome was utilisation of medical services in the public health system in the last 12 months. Structural equation modelling was guided by Andersen’s behavioural model.

**Findings:**

Utilisation of medical services in the last 12 months was reported by 64% of the participants. Lower age (β = -0.138), poor internal housing conditions (β = 0.184), lower frequency of home visits by community health workers (β = -0.129), seeking the same health care service (β = 0.264) and regular use of medication (β = 0.212) directly predicted utilisation of medical services. Significant indirect effects were detected between predisposing (sex, being married, functional dependence), enabling (income, number of goods, external housing conditions, registration in primary care, distance between household and primary care unit), need characteristics (number of chronic diseases) and utilisation medical services in the last 12 months.

**Conclusions:**

The determinants of utilisation of medical services among elderly people living in rural areas are complex and interconnected encompassing demographics, socioeconomic factors, characteristics of the health services, number of chronic diseases and regular use of medication.

**Supplementary Information:**

The online version contains supplementary material available at 10.1186/s12913-025-12945-w.

## Background

The global population is experiencing rapid and unprecedent aging. According to WHO, 17% of the world’s population will be aged 60 years or older by 2030. Moreover, the proportion of people aged 80 years or over is expected to triple between 2020 and 2050 [1]. The current global demographic transition is particularly pronounced in low- and middle-income countries (LMICs), where the pace of ageing is faster than in high-income countries. Recent predictions suggest that 67% of the world’s population over 60 years will live in LMICs by 2050 [2]. The population aging process has accelerated in Brazil since the 1960s. According to the latest demographic census in Brazil in 2022, about 32 million people were aged 60 years or older, representing 15.6% of the total population. This figure corresponds to an increase of 56.0% in elderly population compared to 2010, when the number of people in this age group was over 20.5 million [3].

The population ageing phenomenon has substantial impacts on the country’s health and social public services, demanding a reorganisation of health services to meet the health care needs of elderly people. Health care systems are still failing to meet the expectations and needs of older people, whose situation is considered worse in LMICs due to the double burden of non-communicable and communicable diseases [4]. In addition, disparities in the utilisation of health care among age groups have been reported, suggesting that elderly people face greater barriers to health care, such as lack of information about available health care units, transportation costs, shortage of geriatricians and negative attitudes and behaviours of service providers [4–6].

Public health services in Brazil are provided by the Unified Health System (Sistema Único de Saúde - SUS), which principles encompass universal, equitable and comprehensive access to primary, secondary and tertiary health care for all age groups [7]. Data from recent National Health Surveys in Brazil suggested that people aged 60 years and older were the ones who most sought out and used health services in the SUS [8, 9]. However, there were significant geographical inequalities in health care utilisation among elderly people in Brazil [8, 9]. The prevalence of medical consultations during the last year in rural and urban areas was 81.8% and 90.5%, respectively [10]. Moreover, elderly people living in rural areas were 39% less likely to have a medical visit within the last year than those living in urban areas [10].

The Behavioural Model of Health Services Use, developed by Andersen, has been widely used to investigate the determinants of health service use [11, 12]. The model acknowledges that people’s health service experiences can be described according to three major components: predisposing, enabling and need factors. Predisposing factors refer to pre-existing conditions before the onset of illness, such as demographic characteristics, education and social relationships. Enabling factors are organisational and financial conditions, including income, health insurance and regular sources of health services. Need factors can be differentiated into perceived need (e.g. people’s health experiences) and evaluated need (professional assessment) [12]. Andersen’s behavioural model has served as a theoretical model to investigate the predictors of health services for various health problems, such as mental health and breast cancer, across different levels of health care and age groups [12–15]. However, the vast majority of research has been carried out in the US, Canada and Europe [12]. Studies assessing the determinants of health services utilisation in rural areas in South America are scarce.

Rural areas in Brazil are characterised by population sparseness, diverse cultural traditions, isolation, and large distances between people’s houses and public health services [16, 17]. In the Brazilian Amazon region, poor socioeconomic and sanitation conditions, low educational attainment and limited access to health care pose important barriers to population’s health [17, 18]. This study sought to examine the predisposing, enabling and need factors associated with utilisation of medical services in the public health system among community-dwelling elderly people living in a rural community of the Brazilian Amazon region.

## Methods

### Study design and population

A household-based cross-sectional study was conducted in the rural community “Vila de Novo Remanso”, a rural village located on the left bank of the Amazon River, city of Itacoatiara, Amazonas, Brazil. The city is 216 Km from Amazonas state capital Manaus. The population size of rural community “Vila de Novo Remanso” was 4,950 habitants living in 1,876 households in 2022 [19].

All households in the Novo Remanso village were visited, and every resident aged 60 years or older was invited to participate in the study. Older adults who agreed to participate and signed the informed consent were interviewed. Individuals who had resided in the community for less than 30 days or who did not achieve the minimum score on the Verbal Fluency Test were excluded, as they were considered unable to provide accurate responses during the interview.

Elderly people were initially individually interviewed at their households without support from relatives and the interviewer to assess their cognitive function. The cognitive test was administered by the main study interviewers after obtaining consent to participate in the study and prior to the main study data collection interviews. Eligible participants were excluded if they did not achieve a minimum score of 9 in the cognitive test according to the Verbal Fluency Test [20].

Initially, the target population was estimated by the fieldwork research team through household visits in all 13 census tracts using the maps from the Brazilian Institute of Geography and Statistics (IBGE). A total of 371 elderly people residents were identified and invited. Of them, 302 elderly people agreed to participate (response rate = 76.8%). Sixteen individuals with cognitive impairment and one with incomplete data were excluded. The studied sample comprised 285 elderly people.

### Data collection and sample size calculation

Data were collected at participants households through individual interviews conducted by two previously trained postgraduate students in public health (GSBA and RDSP) using a structured questionnaire. Data collection occurred from August to October 2023 and consisted of demographics, socioeconomic characteristics, housing conditions, individual health care characteristics, frailty, and health status.

The questionnaire’s items were gathered from the Brazilian National Health Survey (NHS), a nationwide home-based survey and The Study of Health, Well-Being, and Aging [21, 22]. The questionnaire was filled out using smartphones and the Research Electronic Data Capture (REDCap) application that allowed fieldwork data collection without internet access and registered the geolocation of participant’s household. A pilot study was conducted with ten elderly people from the same rural community selected by convenience who did not participate in the main study. The average application time, and the clarity and understanding of the questionnaire items were assessed. Data collected in the pilot study were not included in the database analysed.

A study with 285 participants would lend a power of 90% to estimate a structural equation model involving 15 observed variables and 4 latent variables considering a significance level of 5% to detect statistically significant effects of 0.24 [23]. The study power for structural equation modelling was calculated using the statistical calculator available on https://www.danielsoper.com/statcalc.

### Utilisation of medical services in the public health system

Medical services utilisation in the public health system during the last 12 months was measured according to the following item: ‘Have you visited a medical doctor in the public health services in the last 12 months?’. Response options were 0 = no, 1 = yes. The question referred to medical visits in public primary health services, fluvial health care unit, specialised health services or household medical visits. Participant’s utilisation of emergency services was not considered.

### Measures

The independent variables were grouped into predisposing (six variables), enabling (eight variables) and need (four variables) characteristics according to Andersen’s Behavioural Model of Health Services Utilisation [24].

Predisposing characteristics included age, sex (1 = male, 2 = female), skin colour, married/living with partner (1 = no, 2 = yes), education (number of school years completed with approval), and functional dependence. Skin colour was assessed according to self-reported skin colour based on the categories proposed by the IBGE: white, pardo/brown, black, yellow, and indigenous [25]. Functional dependence was a latent variable assessing different functional limitations in performing daily life activities using the following indicators: ‘mobility impairments’, ‘difficulties in dressing independently’ and ‘difficulties in bathing oneself’. These items were responded on a 4-point Likert scale: 1 = “profound difficulty”, 2 = “severe difficulty”, 3 = “minor difficulty” and 4 = “no difficulty” [22].

Enabling characteristics were monthly *per capita* income, number of goods in the household, external housing conditions, internal housing conditions, registration in primary care in the Brazilian public health system (1 = no, 2 = yes), frequency of home visits by community health workers in the last 12 months (1 = monthly, 2 = every 2 months, 3 = between 2 and 4 months, 4 = once, 5 = never), distance in meters between household and primary care unit (assessed using the geolocation of participant’s household and primary care), usually seek the same healthcare services (1 = no, 2 = yes). Monthly *per capita* income was calculated dividing the total amount of income of the family members residing in the household the previous month, including wages, pensions and any social cash transfer program, by the number of residents. Number of goods was a latent variable manifested in five indicators: number of freezers, microwaves, air conditioners, washing machines, and televisions. External housing conditions was a latent variable assessed using four indicators: type of pavement of the street of the household (1 = asphalt, 2 = other, 3 = dirt or gravel), drinking water supply in the household (1 = bottled mineral water, 2 = piped water, 3 = artesian water well), water faucet in the house (1 = yes, 2 = no), and household garbage collection (1 = regular collection at home; 2 = drop-off location in the community; 3 = buried or burned in the community). Internal housing conditions was a latent variable measured by three indicators: floor covering (1 = ceramic, 2 = concrete, 3 = wood), location of household’s toilet (1 = inside the house, 2 = outside the house), and number of toilets.

Need characteristics included number of chronic diseases, regular use of medication (1 = no, 2 = yes), bedridden in the last two weeks (1 = no, 2 = yes) and self-rated health. Number of chronic diseases was assessed based on medical diagnosis of diabetes, cardiovascular diseases, cancer, osteoporosis, dyslipidaemia, rheumatological diseases, chronic renal diseases, and depression. Self-rated health was assessed using the question: ‘In general, how would you rate your health status?’ (1 = very good; 2 = good, 3 = regular, 4 = poor, 5 = very poor).

### Theoretical model

**Fig. 1:**
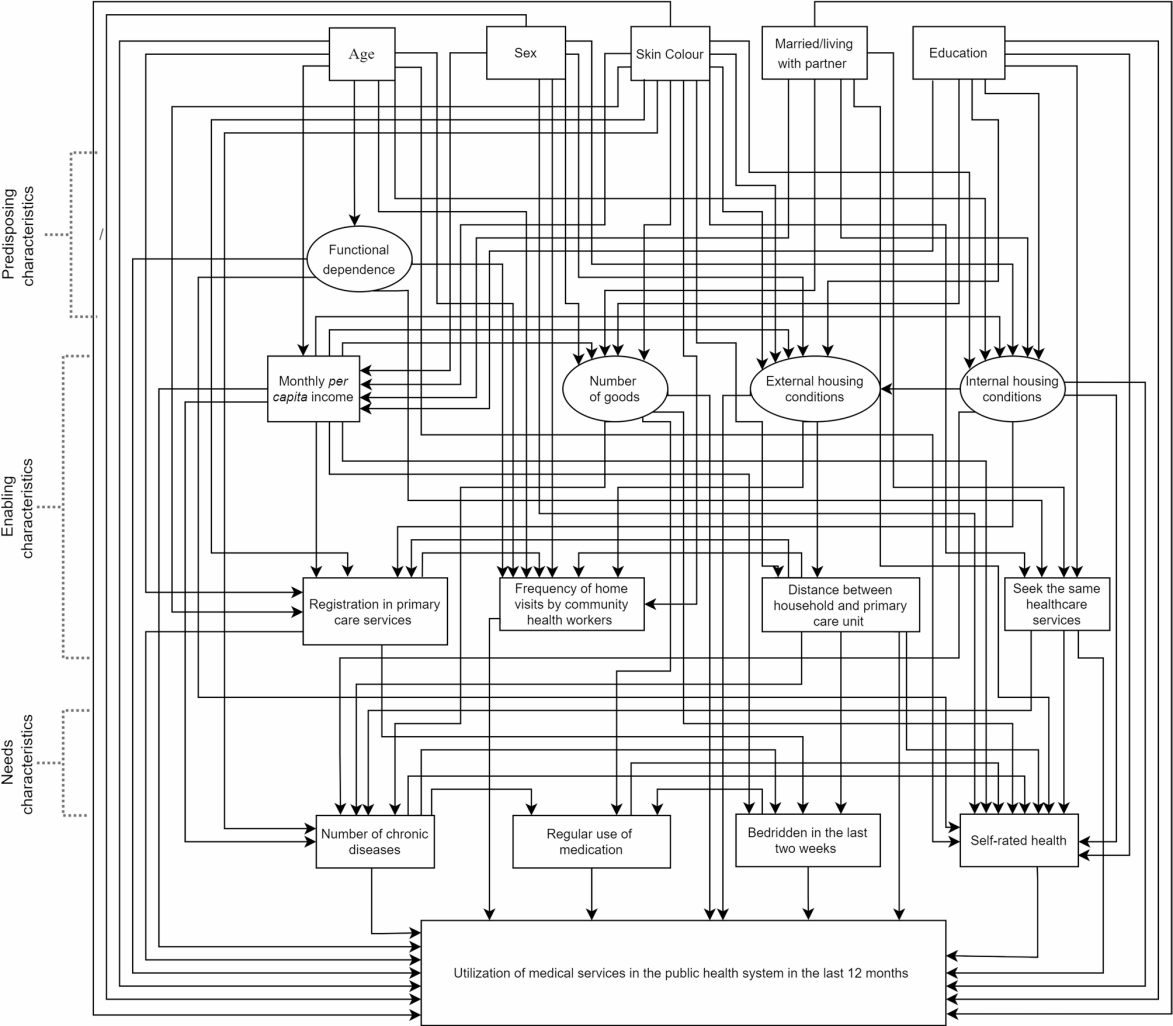
Hypothesised theoretical model on relationships between predisposing, enabling and need characteristics and frequency of utilisation of medical services in the public health system during the last 12 months

The theoretical model of the present study was based upon the Andersen behavioural model [24] (Fig. 1). According to Andersen’s model, the determinants of health services utilisation can be grouped into three categories: (1) predisposing factors, including demographics and social structure characteristics; (2) enabling factors, encompassing logistical and financial resources and the organisation of the health system; and (3) need factors, including both individual perceived health status and normative health needs assessed by health professionals. These factors interact to explain health services utilisation. According to this model, it was hypothesised that demographics, education and functional dependence (predisposing characteristics) and enabling characteristics (income, housing conditions and health services factors) would influence need characteristics (number of chronic diseases, regular use of medication, bedridden in the last two weeks and self-rated health) and utilisation of medical services in the public health system. In addition, it was conjectured that enabling and need characteristics would mediate the association between predisposing characteristics and medical services in the public health system.

### Data analysis

The independent variables were described using means and 95% confidence intervals (CIs) (continuous variables) and frequency and 95% CIs (categorical variables) according to utilisation of medical services in the public health system in the last 12 months.

Confirmatory factorial analysis (CFA) was used to assess the measurement model and the corresponding indicators of the four latent variables: functional dependence, number of goods, external housing conditions, and internal housing conditions. Factor loadings, 95% CIs and *p* values were estimated [26].

Direct and indirect associations between observed and latent variables were tested using structural equation modelling (SEM) according to the Andersen’s behavioural model [24] (Fig. 1). The standardised direct effects represent a direct path from one variable to another, and standardised indirect effects indicate a pathway between two or more variables mediated by another variable. The maximum likelihood method via bias-corrected bootstrap with 900 resampling from the original data set was used to estimate direct and indirect effects and 95% CIs [27]. Mediation was assessed by testing the statistical significance of the indirect effects. The following fit indices and thresholds were employed to evaluate the adequacy of the measurement and structural models: χ²/df < 3.0, comparative fit index (CFI) ≥ 0.90, goodness of fit index (GFI) ≥ 0.90, standardised root mean squat residual (SRMR) ≤ 0.08, and root mean square error of approximation (RMSEA) ≤ 0.06 [28]. The regression weights and fit indices of the full model were initially assessed. Then, nonsignificant direct paths were removed, and the model was re-estimated to obtain a statistically parsimonious model. Descriptive analysis was performed using IBM SPSS Statistics for Windows, version 21 (IBM Corp., Armonk, NY, USA). CFA and SEM were conducted using SPSS AMOS 24.0 software. The significance level established for all analyses was 5% (*p* ≤ 0.05).

## Results

The frequency of utilisation of medical services in the public health system during the last 12 months among the elderly residents of Vila de Novo Remanso was 63.9% (95%CI: 58.1–69.3). Predisposing, enabling and need characteristics are presented according to utilisation of medical services in the public health system in Table 1. The mean age of participants was 71.8 years old, ranging from 60 to 94 years, and nearly half of them were females (48.8%). Most of the participants were of pardo/brown skin colour (71.9%), married or living with partner (60.4%) and did not report mobility impairment (98.2%). Education and income were predominantly low. The average years of schooling was 4.1 (15.1% illiterate) and mean of monthly *per capita* income was 1,112.60 Brazilian reais. Nearly 84% of participants were registered in the primary care services and 74% reported having usual source of care, mainly in primary care itself. More than 70% of the sample reported one or more chronic diseases and 62.5% regularly use medication. Nearly 33% of the participants reported good and very good health.

**Table 1:** Distribution of predisposing, enabling and need characteristics among elderly people living in a rural community of the Brazilian Amazon region

Variables		Total Mean/% (95% CI)	Utilisation of medical services in the public health system in the 12 last months	
			No Mean/% (95% CI)	Yes Mean/% (95% CI)
Predisposing characteristics				
Age		71.8 (70.8–72.8)	73.2 (71.4–75.0)	71.0 (69.9–72.1)
Sex	Male	51.2 (45.4–57.0)	47.6 (38.1–57.2)	53.3 (46.0–60.5)
	Female	48.8 (42.0–54.6)	52.4 (42.8–61.9)	46.7 (39.5–54.0)
Skin colour	White	15.1 (11.4–19.8)	11.7 (6.7–19.5)	17.0 (12.2–23.2)
	Padro/brown	71.9 (66.4–76.9)	75.7 (66.5–83.1)	69.8 (62.7–76.0)
	Black	7.0 (4.6–10.6)	11.6 (6.7–19.4)	4.4 (2.2–8.6)
	Indigenous	4.2 (2.4–7.3)	1.0 (0.1–6.6)	6.0 (3.4–10.6)
	Not declared	1.8 (0.7 – 4.2)	-	2.8 (1.1. –6.5)
Married/living with partner	No	39.6 (34.1–45.5)	43.7 (34.4–53.4)	37.4 (30.6–44.6)
	Yes	60.4 (54.5–65.9)	56.3 (45.6–65.6)	62.6 (55.4–69.4)
Education		4.1 (3.7–4.6)	4.3 (3.5–5.1)	4.0 (3.4–4.5)
* Functional dependence*				
Mobility impairment)	No/minor difficulty	98.2 (95.8–99.3)	96.1 (90.0–98.6)	99.5 (96.2–99.9)
	Profound/severe	1.8 (0.7–4.2)	3.9 (1.5–10.0)	0.5 (0.1–3.8)
Difficulties in dressing up	No/minor difficulty	97.9 (95.4–99.1)	97.1 (91.2–99.1)	98.4 (95.0–99.5)
	Profound/severe	2.1 (0.9–4.6)	2.9 (0.9–8.8)	1.6 (0.5–5.0)
Difficulties in bathing oneself	No/minor difficulty	98.9 (96.8–99.7)	98.1 (92.3–99.5)	99.5 (96.2–99.9)
	Profound/severe	1.1 (0.3–3.2)	1.9 (0.5–7.5)	0.5 (0.1–3.8)
Enabling characteristics				
Monthly *per capita* income (BRL)		1112.6 (978.5–1246.6)	1398.8 (1074.6–1722.9)	950.6 (852.4–1048.6)
Number of goods		3.1 (2.9–3.3)	3.2 (2.7–3.6)	3.1 (2.8–3.3)
* External housing conditions*				
Type of pavement of the street of the household	Asphalt	55.1 (49.2–60.8)	56.3 (46.5–65.7)	54.4 (47.1–61.5)
	Other	4.2 (2.4–7.3)	1.9 (0.5–7.5)	5.5 (3.0–9.9)
	Dirt or gravel	40.7 (35.1–46.5)	41.8 (32.5–51.6)	40.1 (33.2–47.4)
Drinking water supply in the household	Bottled mineral water	24.6 (19.9–29.9)	31.1 (22.8–40.7)	20.9 (15.6–27.4)
	Piped water	73.3 (67.9–78.2)	66.0 (56.3–74.6)	77.5 (70.8–83.0)
	Artesian water well	2.1 (0.9–4.6)	2.9 (0.9–8.8)	1.6 (0.5–5.0)
Water faucet in the house	No	4.6 (2.7–7.7)	5.8 (2.6–12.5)	3.8 (1.8–7.9)
	Yes	95.4 (92.3–97.3)	94.2 (87.5–97.4)	96.2 (92.1–98.2)
Household garbage collection	Regular collection at home	87.4 (83.0–90.8)	84.5 (76.0–90.3)	89.0 (83.5–92.8)
	Drop-off location in the community	4.6 (2.7–7.7)	2.9 (0.9–8.8)	5.5 (3.0–9.9)
	Buried or burned in the community	8.0 (5.1–11.9)	12.6 (7.4–20.6)	5.5 (3.0–9.9)
* Internal housing conditions*				
Floor covering	Ceramic	63.5 (57.7–68.9)	65.1 (55.3–73.7)	62.7 (55.3–69.4)
	Cement	23.2 (18.6–28.4)	22.3 (15.2–31.5)	23.6 (18.0–30.4)
	Wood	13.3 (9.8–17.8)	12.6 (7.4–20.6)	13.7 (9.4–19.6)
Location of household’s toilet	Inside the house	93.3 (89.8–95.7)	94.2 (87.5–97.4)	92.9 (88.0–95.8)
	Outside the house	6.7 (4.3–10.2)	5.8 (2.6–12.5)	7.1 (4.2–12.0)
Number of toilets	0	1.1 (0.3–3.2)	1.9 (0.5–7.5)	0.6 (0.1–3.8)
	1	57.9 (52.1–63.5)	47.6 (38.0–57.3)	63.7 (56.5–70.4)
	2	34.7 (29.4–40.5)	39.8 (30.7–49.6)	31.9 (25.5–39.0)
	3	6.3 (4.0–9.8)	10.7 (6.0–18.4)	3.8 (1.8–7.9)
Registration in primary care	No	16.5 (12.6–21.3)	15.5 (9.7–23.9)	17.0 (12.2–23.2)
	Yes	83.5 (78.7–87.4)	84.5 (76.1–90.3)	83.0 (76.8–87.8)
Frequency of home visits by community health workers	Monthly	35.4 (30.1–41.2)	29.1 (21.2–38.6)	39.0 (32.2–46.3)
	Every 2 months	11.3 (8.0–15.5)	11.6 (6.7–19.4)	11.0 (7.2–16.5)
	Between 2–4 months	13.3 (9.8–17.8)	14.6 (9.0–22.8)	12.6 (8.5–18.3)
	Once	12.6 (9.2–17.0)	13.6 (8.2–21.7)	12.1 (8.1–17.7)
	Never had a visit	27.4 (22.5–32.9)	31.1 (22.8–40.7)	25.3 (19.5–32.1)
Distance between household and primary care unit (meters)		745.2 (690.1–800.4)	807.4 (701.8–913.0)	710.0 (647.5–772.6)
Usually seeks the same health care services	No	26.0 (21.9–31.4)	42.7 (33.5–52.5)	16.5 (11.8–22.6)
	Yes	74.0 (68.6–78.8)	57.3 (47.5–66.5)	83.5 (77.4–88.3)
Need characteristics				
Number of chronic diseases	None	27.3 (22.5–32.9)	35.9 (27.2–45.7)	22.6 (17.0–29.2)
	1	26.0 (21.2–31.4)	22.3 (15.2–31.5)	28.0 (21.9–35.0)
	2	20.7 (16.4–25.8)	17.5 (11.2–26.2)	22.5 (17.0–29.2)
	3 or more	26.0 (21.2–31.4)	24.3 (16.9–33.6)	26.9 (20.9–33.9)
Regular use of medication	No	37.5 (32.1–43.3)	47.6 (38.1–57.2)	31.9 (25.5–39.0)
	Yes	62.5 (56.7–67.9)	52.4 (42.8–61.9)	68.1 (61.0–74.3)
Bedridden in the last two weeks	No	95.1 (91.9–97.1)	95.1 (88.8–98.0)	95.1 (90.7–97.4)
	Yes	4.9 (2.9–8.1)	4.9 (2.0–11.17)	4.9 (2.6–9.3)
Self-rated health	Very good	7.4 (4.8–11.1)	8.8 (4.6–16.0)	6.6 (3.8–11.3)
	Good	25.3 (20.5–30.7)	25.2 (17.8–34.5)	25.3 (19.5–32.1)
	Regular	55.1 (49.3–60.8)	56.3 (46.6–65.6)	54.4 (47.2–61.5)
	Poor	10.5 (7.5–14.7)	8.7 (4.6–16.)	11.5 (7.6–17.1)
	Very poor	1.7 (0.7–4.2)	1.0 (0.1–6.6)	2.2 (0.8–5.7)

*BRL* Brazilian Reals

**Fig. 2:**
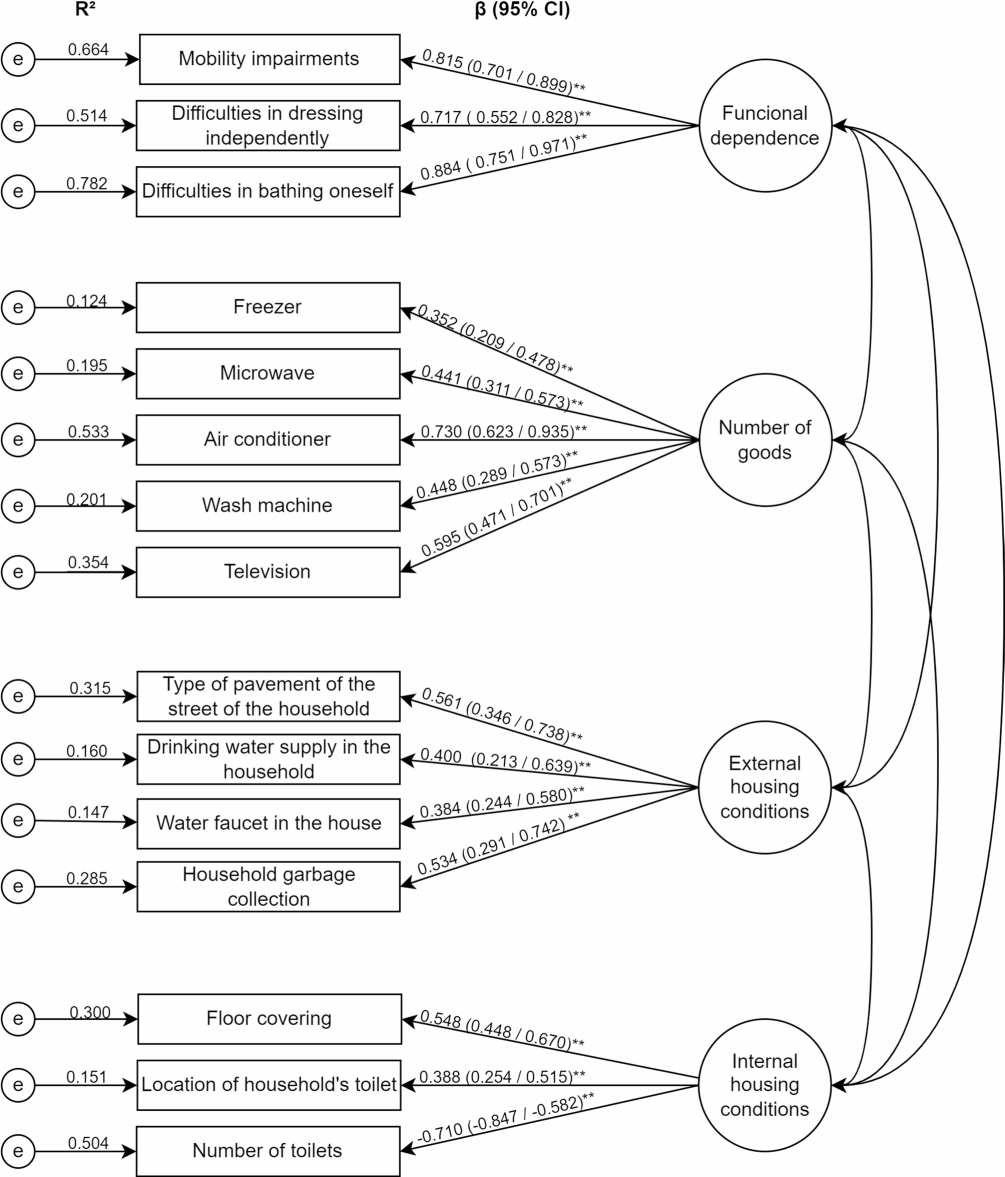
Confirmatory factor analysis of the 4-factor and 15 items measurement model obtained through bootstrap item loadings. β: standardised coefficients (95% confidence intervals). * *P* < 0.05, ** *P* < 0.01

The measurement model assessed using CFA confirmed the presence of individual latent factors: functional dependency, number of goods, external housing conditions, and internal housing conditions (Fig. 2).

The hypothesised model was supported by SEM according to the model fit indices χ²/df = 1.368, GFI = 0.911, CFI = 0.933, SRMR = 0.057, RMSEA = 0.036. The regression weights indicated that neither skin colour nor bedridden in the last two weeks correlated with any variables. Thus, these variables and non-significant direct paths were removed to enhance statistical parsimony. The measurement, full and parsimonious models showed acceptable fit (Table 2).

**Table 2:** Fit indices for the confirmatory analysis of measurement, full and parsimonious models

Model	CMIN/DF	GFI	CFI	SRMR	RMSEA
Measurement	1.817	0.936	0.920	0.060	0.054
Full	1.368	0.911	0.933	0.057	0.036
Parsimonious	1.385	0.903	0.925	0.065	0.037

**Fig. 3:**
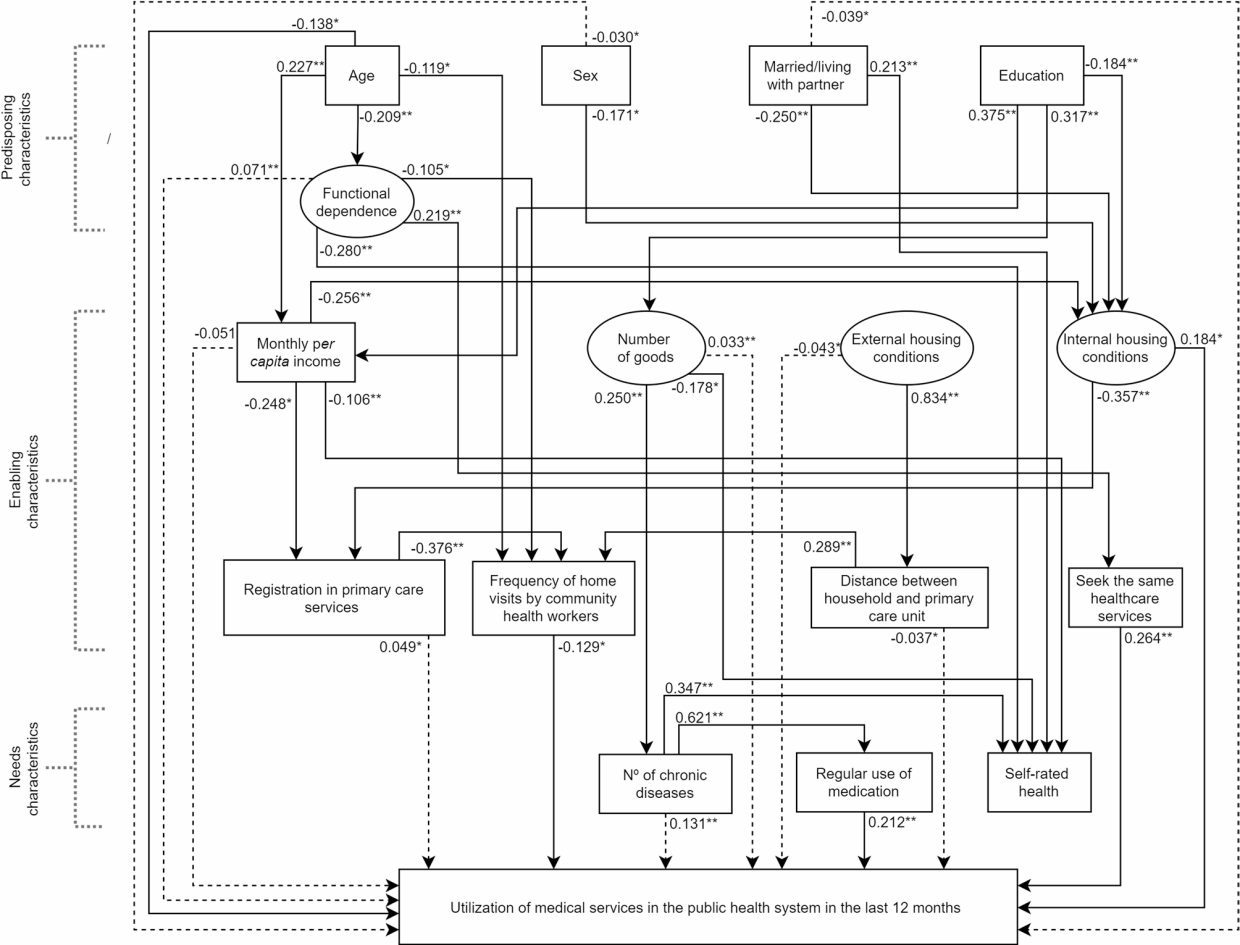
Parsimonious model of the associations between predisposing, enabling and need characteristics and frequency of utilisation of medical services in the public health system during the last 12 months. Solid lines indicate standardised direct effects. Dashed lines indicate standardised indirect effects. Only indirect effects for utilisation of medical services are presented. All estimated indirect effects are described in Table 4. * *P* < 0.05; ** *P* < 0.01

The direct and indirect effects estimated in the parsimonious model are summarised in Fig. 3; Tables 3 and 4. Direct paths showed being younger (β = -0.138) and living in households with poor internal conditions (β = 0.184) were linked to utilisation of medical services in the public health system in the last 12 months. Lower frequency of home visits by community health workers (β = -0.129), seeking the same healthcare services (β = 0.264), and regular use of medication (β = 0.212) were also linked to utilisation of medical services in the public health system in the last 12 months.

**Table 3:** Standardised direct effects of the parsimonious model

Variables	β	Bias-corrected 95% CI	*p*-value
Use of medical services in the public health system			
Age → Use of medical services	-0.138	-0.250 / -0.024	0.017
Internal housing conditions → Use of medical services	0.184	0.031 / 0.323	0.023
Frequency home visits by community health workers → Use of medical services	-0.129	-0.242 / -0.015	0.034
Usually seeks the same health care services → Use of medical services	0.264	0.150 / 0.387	0.002
Regular use of medication → Use of medical services	0.212	0.102 / 0.319	0.002
Regular use of medication			
Number of chronic diseases → Regular use of medication	0.621	0.561 / 0.676	0.002
Usually seeks the same health care services			
Functional dependence → Usually seeks the same health care services	0.219	0.084 / 0.352	0.003
Home visits by community health workers			
Age → Home visits by community health workers	-0.119	-0.226 / -0.012	0.033
Functional dependence → Home visits by community health workers	-0.105	-0.209 / -0.009	0.033
Registration in primary care → Home visits by community health workers	-0.376	-0.481 / -0.284	0.002
Distance between household and primary care unit → Home visits by community health workers	0.289	0.175 / 0.397	0.002
Internal conditions of housing			
Sex → Internal conditions of housing	-0.171	-0.312 / -0.039	0.009
Married/living with partner → Internal conditions of housing	-0.250	-0.420 / -0.088	0.002
Education → Internal conditions of housing	-0.184	-0.319 / -0.037	0.008
Monthly *per capita* income → Internal conditions of housing	-0.256	-0.365 / -0.142	0.002
Functional dependence			
Age → Functional dependence	-0.209	-0.404 / -0.060	0.004
Registration in the Family Health Strategy			
Monthly *per capita* income → Registration in primary care	-0.248	-0.416 / -0.045	0.009
Internal conditions of housing → Registration in primary care	-0.357	-0.500 / -0.230	0.002
Number of chronic diseases			
Number of goods → Number of chronic diseases	0.250	0.095 / 0.401	0.002
Distance between household and primary care unit			
External housing conditions → Distance between household and primary care unit	0.834	0.733 / 0.919	0.002
Monthly *per capita* income			
Age → Monthly *per capita* income	0.227	0.122 / 0.346	0.002
Education → Monthly *per capita* income	0.375	0.226 / 0.502	0.002
Number of goods			
Education → Number of goods	0.133	0.175 / 0.458	0.002
Self-rated health			
Married/living with partner → Self-rated health	0.213	0.111 / 0.311	0.002
Functional dependence → Self-rated health	-0.280	-0.393 / -0.153	0.002
Monthly *per capita* income → Self-rated health	-0.106	-0.195 / -0.036	0.002
Number of goods → Self-rated health	-0.178	-0.338 / -0.027	0.021
Number of chronic diseases→ Self-rated health	0.347	0.244 / 0.441	0.002

**Table 4:** Standardised indirect effects of the parsimonious model

Variables	Use of public health services		
	β	95% CI	*p*-value
Use of medical services in the public health system			
Sex → Use of medical services	-0.030	-0.074 / -0.003	0.025
Married/living with partner → Use of medical services	-0.039	-0.087 / -0.001	0.047
Functional dependence → Use of medical services	0.071	0.026 / 0.130	0.003
Monthly *per capita* income → Use of medical services	-0.051	-0.106 / -0.001	0.043
Number of goods → Use of medical services	0.033	0.010 / 0.067	0.002
External housing conditions → Use of medical services	-0.043	-0.090 / -0.008	0.020
Registration in primary care → Use of medical services	0.049	0.005 / 0.097	0.034
Distance between household and primary care unit → Use of medical services	-0.037	-0.078 / -0.004	0.034
Number of chronic diseases → Use of medical services	0.131	0.062 / 0.199	0.002
Regular use of medication			
Education → Regular use of medication	0.049	0.016 / 0.093	0.002
Number of goods → Regular use of medication	0.156	0.060 / 0.246	0.002
Number of chronic diseases			
Education → Nº chronic diseases	0.079	0.026 / 0.150	0.002
Usually seeks the same health care services			
Age → Usually seeks the same health care services	-0.046	-0.112 / -0.011	0.006
Home visits by community health workers			
External housing conditions → Home visits by community health workers	0.241	0.143 / 0.338	0.002
Internal housing conditions → Home visits by community health workers	0.135	0.079 / 0.206	0.002
Married/living with partner → Home visits by community health workers	-0.034	-0.073 / -0.009	0.002
Sex → Home visits by community health workers	-0.023	-0.051 / -0.005	0.009
Age → Home visits by community health workers	0.035	0.008 / 0.080	0.010
Registration in primary care services			
Per capita income → Registration in primary care	0.092	0.046 / 0.164	0.002
Married/living with partner → Registration in primary care	0.089	0.026 / 0.180	0.002
Sex → Registration in primary care	0.061	0.012 / 0.127	0.009
Internal housing conditions			
Education → Internal housing conditions	-0.096	-0.161 / -0.045	0.002
Age → Internal housing conditions	-0.058	-0.104 / -0.026	0.002
Self-rated health			
Number of goods → Self-rated health	0.087	0.030 / 0.154	0.002
Education → Self-rated health	-0.069	-0.144 / -0.010	0.019

There were several significant indirect effects between predisposing, enabling and need characteristics and utilisation of medical services in the public health system. Utilisation of medical services was indirectly linked to males (β = -0.030), not being married or living with a partner (β = -0.039), lower functional dependence (β = 0.071), lower family income (β = -0.051), greater number of goods (β = 0.033) and better external housing conditions (β =-0.043). Registration in primary care services (β = 0.049), lower distance in meters between household and primary care unit (β =-0.039) and higher number of chronic diseases (β = 0.129) were also indirectly linked to utilisation of medical services in the public health system in the last 12 months. These are the total indirect effects and are made up of a number of specific indirect paths. For instance, the indirect pathway between functional dependency and utilisation of medical services in the public health system can be broken down to two paths: (1) via frequency of home visits by a community health worker, (2) via usually seek the same healthcare services. The distance in meters between household and primary care unit and frequency of home visits by a community health worker mediated the link between external housing conditions and utilisation of medical services in the public health system.

## Discussion

The present study comprehensively examined the relationships between several predisposing, enabling and need characteristics and utilisation of medical services in the public health system among elderly people living in a rural community in the Brazilian Amazon region. Almost 64% of the participants had a medical visit to the public health services during the last 12 months, revealing insufficient levels of medical services utilisation in the population studied. More than 70% of elderly people reported at least one chronic disease, and 62.5% use regular medication suggesting a concerning scenario.

Study findings support the use of the Andersen behavioural model to investigate the determinants of utilisation of health services in elderly people living in rural areas [24]. Even though some of the predictors for health services utilisation might be similar between people living in urban and rural areas (e.g. age, income, self-rated health), environmental and social differences between them suggest the need to consider specific determinants of utilisation of health care among people living in rural areas, such as external and internal housing conditions.

Several significant direct and indirect effects were identified between predisposing, enabling and need characteristics and utilisation of medical services among elderly people living in a rural area of the metropolitan region of Manaus. Age, internal housing conditions, frequency of home visits by community health workers, seeking the same healthcare services, and regular use of medication were direct predictors of medical services utilisation in the public health system in the last 12 months.

Aging is characterised by income decrease and an increase in functional limitations among the elderly people, which in turn are often related to reduced accessibility to health care [29, 30]. However, the results showed that being older was negatively associated with utilisation of medical services. The sample comprised elderly people with low levels of education since 80% had less than 6 years of schooling. This possibly explains the lack of association between education and the utilisation of medical services.

Elderly people with more functional limitations tend to make more medical visits [31, 32]. The greater use of health services among elderly people with functional limitations is possibly explained by the higher prevalence of chronic diseases and comorbidities, and consequently, the higher rates of medication use [32]. According to the study findings, lower functional dependence was indirectly associated with the utilisation of medical services via seeking the same healthcare service, which contradicts the results from previous studies. Thus, in the studied population, being functionally dependent may lead to seeking different healthcare services, which in turn negatively affects the utilisation of medical services.

Enabling characteristics have been considered relevant factors that affect variation in the use of health services [33]. However, there is no consensus on the role of socioeconomic inequalities on utilisation of health services. Elderly people with better socioeconomic conditions are more likely to access healthcare services than those with lower socioeconomic status in countries where individuals must pay for healthcare services, insurance plans or out-of-pocket [33–35]. Social inequalities in health services utilisation are more pronounced in countries with private healthcare systems and developing countries [34, 35]. The four socioeconomic variables investigated in the study were associated with utilisation of medical services. Interestingly, income, number of goods and external housing conditions were indirectly linked to utilisation of medical services among elderly people. Different enabling factors related to health services mediated the above-mentioned links, including registration in primary care services, frequency of home visits by health workers, distance between household and primary care unit and seeking the same healthcare services. These results reinforce the complexity related to the interrelationships between enabling characteristics that influence utilisation of medical services among elderly people living in rural communities.

The outcome of the study considered a medical visit in the public health services because primary health care in the SUS is the main source of access to health care in rural areas in Brazil. Individuals living in rural areas are at greater health risks due to geographical challenges and limited access to healthcare services [36]. These areas also face significant difficulties in attracting and retaining health professionals, especially in the medical doctors [37]. In general, the rural population of the Amazon faces more obstacles in accessing primary health care [17]. Financial and geographical barriers affect the organisation of health care for rural populations, including insufficient availability of appointments, longer waiting times and distance to the service, as well as personal limitations, such as lack of transportation [38]. The elderly population of Brazil has grown significantly over the last decades, resulting in a greater proportion of people with co-morbidities and use of regular medication [3]. Thus, provision of health care to elderly Brazilians living in rural areas has several challenges, which have been identified in the present study.

Frequency of home visits carried out by community health workers was directly associated with the utilisation of medical services and mediated the link between predisposing and enabling characteristics and utilisation of medical services. Home visits of health care professionals facilitate the adequate referral of sick individuals to health services, provide health promotion and prevention, and monitor ongoing treatment and rehabilitation. This is particularly important for elderly people as this age group has higher healthcare needs, requires higher levels of care, and frequently need support to access health services due to long distances [39–41]. Despite their importance, previous studies revealed that home visits by community health workers have declined in recent years in Brazil, which may be related to shortage of these professionals in health teams and territories. In addition, older adults with chronic diseases, low income, and living in economically disadvantaged regions, such as the Northeast Region of Brazil, were less likely of receiving a home visit [40, 42].

Among the need characteristics investigated, regular use of medication was directly related to utilisation of medical services and mediated the link between the number of chronic diseases and utilisation of medical services in elderly people living in rural areas. The use of medication for the management of chronic diseases is common in elderly people. In addition, receiving the diagnosis of at least one chronic disease is a determining factor for the use of health services in this age group regardless of income and education [43–45]. The prevalence of multimorbidity among adults and elderly people is substantial in Brazil and has been associated with the use of health services [46]. Therefore, health promotion and preventive actions are needed to tackle chronic diseases in elderly people living in rural areas.

Previous research conducted in LMICs has reported similar barriers to the use of health services. Lower education, poor socioeconomic status, perception of low severity of disease, and unavailability of accessible health care facilities were the main determinants of non-utilisation of health services among elderly people in rural areas of India [47, 48]. A recent systematic review reported that the lack of universal health coverage was related to excessive out-of-pocket health expenditure in sub-Saharan Africa. In addition, living in rural areas, having an elderly person in the household, and chronic diseases were also associated with excessive out-of-pocket health expenditure, further impoverishing the poorest households in sub-Saharan Africa and leading to unmet health care needs [49]. Another systematic review of predictors of utilisation of antenatal care services in Ethiopia revealed the socioeconomic barriers of antenatal care use, including rural residence, and poor women or husband education [50].

The use of a representative and random sample of elderly people indicates that study findings could be relevant to other rural areas with similar socioeconomic conditions. The structural equation modelling was a robust statistical method to test the proposed theoretical model and to explore the simultaneous relationships between the predictors of utilisation of medical services. However, some limitations should be acknowledged. First, the cross-sectional design imposes limitations in the assessment of causality on the reported associations. Second, information directly obtained from elderly people is prone to recall bias. Finally, the utilisation of medical services was not verified with the healthcare units, relying on participants’ information.

## Conclusion

The utilisation of health services in the last 12 months by the elderly people living in an Amazonian rural community was insufficient. Demographics, socioeconomic factors, characteristics of the health services, chronic diseases and regular use of medication were relevant predictors for the utilisation of medical services in this population. Study findings also suggest that enhancing home visits by community health workers and favouring the use of the same healthcare services may improve the use of medical services in the public health system among elderly people living in rural areas.

## Supplementary Information

Supplementary Material 1.

## Data Availability

The datasets used and analyzed during the current study are available from the corresponding author on reasonable request.
