# Supplementary material for: Barriers and facilitators to accessing healthcare services among elderly people living in a rural Amazonian community, Brazil
Source: BMC Health Serv Res. 2025 Jul 2;25:886. doi: 10.1186/s12913-025-12945-w (PMC12225115; doi:10.1186/s12913-025-12945-w)
Supplement: Supplementary file 1 — Supplementary Material 1. [file 12913_2025_12945_MOESM1_ESM.pdf]

Questionnaire

Household Identification:
(interviewer and household code) ______________________

Questionnaire identification

Interview date: ______________________

Georeferencing coordinates of the home.

Latitude: ______________________

Longitude: ______________________

Interviewer
- ☐ Gleica
- ☐ Rita
- ☐ Other

Specify ______________________

Name: ______________________

Census Sector
- ○ 02
- ○ 03
- ○ 04
- ○ 23
- ○ 24
- ○ 29
- ○ 30
- ○ 31
- ○ 32
- ○ 33
- ○ 42
- ○ 43
- ○ 44
- ○ 53

Semantic Verbal Fluency (Animals) ______________________

Semantic Verbal Fluency (Fruits) ______________________

Part B: Characteristics of the interviewee and family

B1: Sex:

◯ masculine
◯ feminine

Date of birth:

____

Age:

____

☐ Does not know how to inform

Age provided:

____
(in full years)

B3: What is your color or race:

◯ White ◯ Black ◯ Yellow (origin oriental: Japanese, Chinese, Korean, etc.)
◯ Brown ◯ Indigenous ◯ I didn't know inform
(Read the answer options)

B4: Can [name] read or write?

◯ Yes ◯ No

B5: Years of study completed by [name] with approval:

____
(Put 0 if you never went to school)

B6: What is the highest level of education among the residents of the household?

◯ Incomplete Basic Education
◯ Complete Basic Education
◯ Incomplete secondary education
◯ Complete Secondary Education
◯ Incomplete Higher Education
◯ Complete Higher Education
◯ Did not respond

B6_2: Kinship in relation to the elderly

◯ Spouse
◯ Son (daughter)
◯ Genre
◯ daughter-in-law
◯ Brother/sister
◯ Nephew (a)
◯ Grandson (a)
◯ The elderly man himself
◯ Other

Specify

____

B7: How many people including [name] live in your home?

____
(Dial 999 for "don't know/no answer")

B8: What is your main occupation?	◯ Agriculture/Fish farming/Extractivism - Self-Employed Owner ◯ Agriculture/Fishing height/Extractivism - Employee<br>◯ Activity related to tourism - Self-employed<br>◯ Tourism-related activity - Employee<br>◯ Trade/service activity - Self-employed<br>◯ Trade/service activity - Employee<br>◯ Civil servant ◯ From home<br>◯ Student ◯ Retiree<br>◯ Unemployed ◯ Other<br>◯ Did not respond<br>(Note: if you have more than one occupation, register the reported as main.)
Specify:	______________________
B9: [name], do you live with a spouse or partner?	◯ Yes<br>◯ No
B10: How long has your family lived here? community?	______________________<br>(In years)
age = [b2_age] ([b2_1_age_inf])	
B11: Your house is:<br>◯ Own<br>◯ Rented<br>◯ Lent / Borrowed	
Observe and answer questions B12 to B14.	
B12: Floor type:	◯ Dirt floor<br>◯ Wood<br>◯ Ceramics<br>◯ Cement<br>◯ Other<br>◯ IGN
B13: Wall type:	◯ Straw ◯ Wood ◯ Brick<br>◯ rammed earth/clay ◯ Canvas/plastic<br>◯ Other ◯ IGN
B14: Type of covering/roof:	◯ Straw ◯ Wood ◯ Slab<br>◯ Canvas/plastic ◯ Clay tile<br>◯ Zinc or asbestos tile<br>◯ Other ◯ IGN
B15: [name] How many rooms are there in your house?	______________________<br>(Dial 999 for "don't know/no answer")

B16: What is the main source of water supply for this household?

- General distribution network
- Deep or artesian well
- Shallow well, phreatic well or waterhole
- Source or spring
- River, lake or stream
- Stored rainwater
- Other (specify)
- IGN

B16.1: Specify

____

B17: Does the water used in this household come through pipes?

- Yes, in at least one room
- Yes, only on the land/property
- It doesn't arrive channeled
- Don't know
- Did not respond

B18: Is there at least one tap that works (with water) INSIDE the house?

- Yes
- No
- Did not respond

B19: Where does the water that the family uses to DRINK come from?

- Local network
- Rain collection
- Artesian well
- Shallow well (Cacimba)
- River, stream, lake
- Other
- Industrialized Mineral
- Did not respond

B20: Does [name] treat the water used for drinking?

	Yes	No	IGN
B20.1: Treated with hypochlorite	○	○	○
B20.2: Filtered/boiled	○	○	○
B20.3: Other type of treatment	○	○	○

Specify

____

B21: How many bathrooms (with shower and toilet) are there in this household?

____

B22: The place where residents usually defecate is:

- Inside the house (latrine/toilet)
- Outside the home (latrine/toilet used only by the household)
- Outside the home (latrine/communal toilet)
- In the bush
- Other
- Did not respond

Specify:

____

B23: IF THERE IS A LATRINE/TOILET, where does the waste go?

- ◯ Septic tank
- ◯ Rudimentary/shallow pit
- ◯ Straight to the river/stream
- ◯ Open sky
- ◯ Other
- ◯ Did not respond

Specify: ______________________________

B24: What is done with the waste produced in the domicile?

- ◯ Collected by cleaning service directly at home
- ◯ Placed in location in community for later collection by service cleaning
- ◯ Buried or burned in community
- ◯ Buried or burned outside of the community
- ◯ Thrown into a river or lake
- ◯ Thrown on vacant land
- ◯ Other
- ◯ IGN

Specify: ______________________________

B25: Does this home have electric lighting?

- ◯ Yes
- ◯ Yes, but discontinuous
- ◯ No
- ◯ Did not respond

B26: Electrical energy comes from:

- ☐ Own generator
- ☐ Generator in the community
- ☐ Public distribution network / electricity for all
- ☐ Other
- ☐ I don't know
- ☐ Did not respond

Specify: ______________________________

B27: For cooking the following is used:

- ☐ Gas
- ☐ Coal or firewood, indoors, with exhaust out
- ☐ Coal or firewood, indoors, without exhaust
- ☐ Coal or firewood outside the home
- ☐ Other:
- ☐ Did not respond

Specify: ______________________________

B28: Considering the section of the street where you live, Would you say the street is:

- ◯ Asphalted/paved
- ◯ Earth/gravel
- ◯ Other
- ◯ I don't know
- ◯ Did not respond

Specify: ______________________________

B29: [name] has a cell phone with network access local telephone service (call, SMS) at home?

- ◯ Yes
- ◯ No
- ◯ Did not respond

B30: Do any residents have access to the internet at home via computer, tablet, mobile phone, television or other equipment?

- ◯ Yes
- ◯ No
- ◯ I don't know
- ◯ Did not respond

B31: What type of internet access is used?

- ☐ Own wifi access
- ☐ Access via mobile data at home (3G, 4G, 5G)
- ☐ Other
- ☐ Don't Know
- ☐ Did not respond

Specify:

____

B32: [name] Do any of the residents of the household work?

- ◯ Yes
- ◯ No
- ◯ Don't know
- ◯ Did not respond

B33: Does [name] receive a pension?

- ◯ Yes
- ◯ No
- ◯ Did not respond

B34: On average, how much did they receive, in reais, together? all the people who live in your house including salaries, family allowance, pension, rent, retirement or other income?

____
(1 Minimum wage: 1320)

B35: How many monthly workers (nanny, cleaning lady, carer, driver...) Does [name] have at home?

____

Part C: Lifestyle

C1: In general, how do you consider your health status:

- ◯ Very good
- ◯ Good
- ◯ Regular
- ◯ Bad
- ◯ Very Bad

(read the options)

C2: Does [name] smoke any tobacco products?

- ◯ Yes, daily
- ◯ Yes, sporadically
- ◯ I don't smoke currently
- ◯ I have never smoked

C3: On average, how many cigarettes do you smoke per day?

- ◯ Less than a wallet
- ◯ A wallet
- ◯ More than one wallet
- ◯ I don't know
- ◯ Did not respond

(1 pack = 20 cigarettes)

C4: If less than one pack, how many cigarettes per pack? day?

____
(99. Don't know / don't remember. 1 wallet = 20 cigarettes)

C5: How old was [name] when he started smoking?

____

C6: How long ago did [name] stop smoking?

____
(in months)

C7: How often does [name] usually consume alcoholic beverages?

- ○ I never drink
- ○ Less than once a month
- ○ Once or more per month
- ○ Did not respond

C8: How many days a week do you usually consume alcoholic beverages?

____
(99. I don't know / I don't remember)

C9: In general, on a day that you drink, how many drinks do you drink?
Does [name] drink alcohol?

____
(999 if you don't know / don't remember)

C10: Do you practice any type of physical activity?

- ○ Yes
- ○ No
- ○ Don't know
- ○ Did not respond

C11: What physical activity does [name] practice?

____

C12: How many times a week do you exercise?
physical?

____
(9. I don't know/ I don't remember)

C13: How long does the practice usually last?
physical activity?

____
(in minutes)

Part D: Health of the Elderly

D01: In general, how difficult is it for [name] to eat alone, with a plate placed in front of him/her, including holding a fork, cutting food and drinking from a cup:

- ○ Can't
- ○ It has great difficulty
- ○ There is little difficulty
- ○ There is no difficulty
- ○ Did not respond

(Read the answer options)

D02: In general, how difficult is it for you to take a bath alone, including getting in and out of the shower or bath:

- ○ Can't
- ○ It has great difficulty
- ○ There is little difficulty
- ○ There is no difficulty
- ○ Did not respond

(Read the options)

D03: In general, how difficult is it for you to go to the bathroom alone, including sitting on and getting up from the toilet?

- ○ Can't
- ○ It has great difficulty
- ○ There is little difficulty
- ○ There is no difficulty
- ○ Did not respond

(Read the options)

D04: How difficult is it for you to dress yourself, including putting on socks and shoes, zipping up and unbuttoning?

- ◯ Can't
- ◯ It has great difficulty
- ◯ There is little difficulty
- ◯ There is no difficulty
- ◯ Did not respond

(Read the options)

D05: How difficult is it for you to walk around the house alone from one room to another, on the same floor (such as from the bedroom to the living room):

- ◯ Can't
- ◯ It has great difficulty
- ◯ There is little difficulty
- ◯ There is no difficulty
- ◯ Did not respond

(Read the options)

D06: How difficult is it for you to lie down or get up from bed alone?

- ◯ Can't
- ◯ It has great difficulty
- ◯ There is little difficulty
- ◯ There is no difficulty
- ◯ Did not respond

(Read the options)

Use of Medications

D07: Does [name] use any medication, which was prescribed by a doctor, for continuous regular use (daily)?

- ◯ No
- ◯ Yes
- ◯ Don't Know
- ◯ Did not respond

D08: Which medications for regular use were prescribed for you to use?

____
(Write down all medications.)

D09: Most of the time, where do you get most of the medications you use?

- ◯ At the UBS that serves the community
- ◯ Elsewhere for free
- ◯ Pay for the medicine
- ◯ Don't know
- ◯ Did not respond

D10: In the last month, has [name] stopped taking any medication that he or she needed? Why?

- ◯ Yes. I didn't adapt to the medication.
- ◯ Yes. I couldn't get it for free.
- ◯ Yes. I thought I didn't need it.
- ◯ Yes. Another reason
- ◯ I didn't stop using any medication
- ◯ Don't Know
- ◯ Did not respond

Specify

____

D11: What are the names of the medications that you are taking? Have you stopped using it?

____

Part E: Chronic Diseases

DC01: Does [name] wear glasses or other assistive devices to deal with vision problems?

- Yes
- No
- Did not respond

DC02: Does [name] have permanent difficulty seeing?

- No, no difficulty
- Yes, some difficulty
- Yes, very difficult
- Can't see at all
- Did not respond

DC03: Does [name] use a hearing aid or other device to hear better?

- No
- Yes
- Did not respond

DC04: Does [name] have permanent hearing difficulties?

- No, no difficulty
- Yes, some difficulty
- Yes, very difficult
- can't hear at all
- Did not respond

DC05: Does [name] use any device to get around (wheelchair, crutch, cane, walker or prosthesis)?

- No
- Yes
- Did not respond

DC06: Does [name] use any assistive devices to perform movements with the upper limbs?

- No
- Yes
- Did not respond

DC07: In the last twelve months, has [name] received any rehabilitation care on a regular basis (physical therapy, occupational therapy, speech therapy, psychotherapy)?

- No
- Yes
- Did not respond

DC08: Does [name] have any chronic spinal problems, such as back or neck pain, low back pain, sciatica, vertebrae or disc problems?

- No
- Yes
- I don't know
- Did not respond

DC09: How old was [name] when he started back problem?

____

DC10: In general, to what extent does the back problem limit your usual activities (working, doing household chores, etc.):

- Does not limit
- A little
- Moderately
- Intensely
- Very intensely
- Did not respond

(Read the options)

DC11: When was the last time [name] had an eye exam with a healthcare professional?

- Less than 6 months ago
- Between 6 months and 1 year
- Between 1 year and less than 2 years
- Between 2 and 3 years ago
- More than 3 years ago
- Never taken an exam
- Did not respond

DC12: Has a doctor ever diagnosed you with cataracts in one or both eyes?	◯ No<br>◯ Yes<br>◯ Don't know / don't remember<br>◯ Did not respond
DC13: Was there an indication for eye surgery to remove cataracts?	◯ No<br>◯ Yes<br>◯ Don't know / don't remember<br>◯ Did not respond
DC14: Did [name] have the surgery?	◯ Yes<br>◯ No
DC15: What is the main reason for not having the surgery?	◯ It's marked, but not done yet<br>◯ Didn't find it necessary<br>◯ Didn't get a spot<br>◯ I was having financial difficulties<br>◯ I was having transportation difficulties<br>◯ The health service was very far away<br>◯ The health plan did not cover the surgery<br>◯ I didn't know where to have the surgery<br>◯ There was no one to accompany him<br>◯ Other<br>◯ Did not respond
Specify	__________________________________
DC16: Your surgery was performed by the public health service	◯ No<br>◯ Yes
DC17: In the past twelve months, has [name] had a flu shot?	◯ No<br>◯ Yes<br>◯ Don't know / remember<br>◯ Did not respond
DC18: What is the main reason [name] did not get the flu vaccine?	◯ Rarely gets the flu<br>◯ I didn't know it was necessary to take the vaccine against the flu<br>◯ I didn't know where to get the vaccine<br>◯ Afraid of the reaction<br>◯ Afraid of injections<br>◯ There was no one to accompany him to the health service.<br>◯ I was having financial difficulties to pay for transportation to the vaccination site<br>◯ Had difficulty finding any means of transport<br>◯ The health service was very far away<br>◯ It was not offered in the service<br>◯ The vaccine was not available<br>◯ Don't believe that the vaccine protects<br>◯ Other<br>◯ Did not respond
Specify	__________________________

DC19: Has [name] had the COVID19 vaccine?

- No
- Yes, one dose
- Yes, two doses
- Yes, three doses
- Yes, four doses Yes,
- five doses

DC20: What is the main reason [name] did not get the COVID-19 vaccine?

- Rarely gets sick
- I didn't know it was necessary to take the Covid vaccine
- I didn't know where to get the vaccine
- Afraid of the reaction
- Afraid of injections
- There was no one to accompany him to the health service.
- I was having financial difficulties paying for transportation to the vaccination site
- Had difficulty getting any means of transport
- The health service was very far away
- It was not offered in the service
- The vaccine was not available
- Don't believe that the vaccine protects
- Other
- Did not respond

Specify

____

DC21: Has a doctor ever diagnosed you with one of the following diseases:

	Yes	No	Did not respond
High blood pressure	○	○	○
Diabetes	○	○	○
Stroke	○	○	○
Cancer	○	○	○
Hypercholesterolemia (high cholesterol)	○	○	○
Osteoporosis	○	○	○
Heart disease (angina, heart attack, insufficiency, arrhythmia)	○	○	○
Asthma (chronic bronchitis)	○	○	○
Arthritis	○	○	○
Rheumatism	○	○	○
Depression	○	○	○
Chronic Renal Failure	○	○	○
Other	○	○	○

Specify:

____

HT01: Do you regularly go to the doctor/health service to monitor your high blood pressure?	◯ Yes, regularly<br>◯ No, only when there is a problem.<br>◯ Never will<br>◯ Did not respond
HT02: What is the main reason why you do not visit the doctor/health service regularly to monitor your blood pressure (high blood pressure)?	◯ The health service is far away or has transportation difficulty<br>◯ The waiting time for service is very long<br>◯ Have financial difficulties to pay for the transport<br>◯ Have financial difficulties to pay for the service<br>◯ Don't think it's necessary<br>◯ The opening hours of the health service is incompatible with your work activities or domestic<br>◯ Don't know who to look for or where to go<br>◯ The health plan does not cover consultations<br>◯ There is no one to accompany him<br>◯ Have transportation difficulties (other than financial)<br>◯ The health service does not have a doctor or not works regularly<br>◯ The pressure is under control<br>◯ Other:<br>◯ Did not respond
Specify	__________________________________
HT03: Has a doctor ever prescribed you any medication for high blood pressure?	◯ Yes<br>◯ No
HT04: In the last two weeks, have you taken medication for high blood pressure?	◯ No, not at all<br>◯ Yes, all of them<br>◯ Yes, some<br>◯ Did not respond
HT05: What is the main reason why you did not take the medication prescribed for high blood pressure?	◯ Unable to obtain it from the public health service<br>◯ Couldn't get the medicine(s) in "Here you have it" "People's Pharmacy"<br>◯ The pharmacy was far away or had difficulty getting there transport<br>◯ Could not find all medicines to buy at the pharmacy<br>◯ I didn't have money to buy<br>◯ Didn't find it necessary<br>◯ You no longer need to take medication because pressure is controlled<br>◯ Other<br>◯ Did not respond
Specify	______________________________
DB01: Do you go to the doctor/health service regularly to monitor your diabetes?	◯ Yes, regularly<br>◯ No, only when there is a problem.<br>◯ No, never go to the doctor for follow-up. diabetes<br>◯ Did not respond

DB02: What is the main reason why you do not visit your doctor/health service regularly to monitor your diabetes?

- ◯ The service is very far away
- ◯ Having difficulty scheduling appointments
- ◯ The waiting time in the health service is very long
- ◯ Have financial difficulties with transportation
- ◯ Have financial difficulties paying for services
- ◯ Don't think it's necessary
- ◯ The health service's opening hours are incompatible with your work or domestic activities
- ◯ The health plan does not cover consultations
- ◯ Don't know who to look for or where to go
- ◯ There is no one to accompany him
- ◯ The health service does not have a doctor or does not operate regularly
- ◯ Diabetes is under control
- ◯ Other:
- ◯ Did not respond

Specify

____

DB03: Has a doctor ever prescribed you any medication for diabetes?

- ◯ Yes, insulin
- ◯ Yes, oral
- ◯ Yes, oral and insulin
- ◯ No
- ◯ Did not respond

DB04: In the last two weeks, because of your diabetes, have you stopped taking any medication?

- ◯ No, none
- ◯ Yes, all of them
- ◯ Yes, only oral ones
- ◯ Yes, just insulin
- ◯ Did not respond

DB05: What is the main reason for not taking the prescribed medication to control diabetes?

- ◯ Unable to obtain it from the public health service
- ◯ Couldn't get the medicine(s) in "Here you have it" "People's Pharmacy"
- ◯ The pharmacy was far away or there was transportation difficulty
- ◯ Couldn't find all the medicines to buy at the pharmacy
- ◯ I didn't have money to buy
- ◯ Didn't find it necessary
- ◯ You no longer need to take medication because your diabetes is under control
- ◯ Other
- ◯ Did not respond

Specify

____

Women's Health

SM01: When was the last time you had a cervical cancer screening test?

- ◯ Less than 1 year ago
- ◯ From 1 year to less than 2 years ago
- ◯ 2 years to less than 3 years ago 3 years
- ◯ or more ago
- ◯ Never did
- ◯ Did not respond

SM02: Why have you never had a preventive exam?

- ◯ Never had sexual relations
- ◯ I don't think it's necessary
- ◯ Shame on you
- ◯ She was never advised to take the exam
- ◯ Don't know who to look for or where to go
- ◯ Have financial difficulties paying for transportation to the service
- ◯ Having difficulty getting transportation
- ◯ Had difficulty making an appointment
- ◯ The waiting time in the health service is very long
- ◯ The health service is very far away
- ◯ The service's operating hours are incompatible with work/domestic activities
- ◯ The health plan does not cover the exam
- ◯ It is scheduled but has not yet been carried out
- ◯ Had surgery to remove the uterus / hysterectomy
- ◯ Other
- ◯ Did not respond

Specify: ______________________________

SM03: Was your last preventive exam for cervical cancer done through the Unified Health System (SUS)?

- ◯ No
- ◯ Yes
- ◯ Don't remember / Don't know
- ◯ Did not respond

SM04: When was the last time a doctor or nurse performed a clinical examination of your breasts?

- ◯ Less than 1 year ago
- ◯ From 1 year to less than 2 years ago
- ◯ 2 years to less than 3 years ago 3 years
- ◯ or more
- ◯ Never did
- ◯ Did not respond

SM05: Has any doctor ever requested a mammogram?

- ◯ No
- ◯ Yes
- ◯ Don't remember
- ◯ Did not respond

SM06: Did you have a mammogram?

- ◯ Yes
- ◯ No

SM07: What is the main reason you did not have a mammogram?

- The exam is scheduled, but you haven't taken the exam yet
- Didn't find it necessary
- I didn't know where to take the exam
- Couldn't score
- I was having financial difficulties
- I was having transportation difficulties
- The waiting time for the mammography service was very long
- The health service with mammography is very far away
- The mammography service's opening hours were incompatible with your work or household activities
- The health plan did not cover the mammogram
- It was never requested by the health professional
- Other:
- Did not respond

Specify

____

SM08: When was the last time you had a mammogram?

- Less than 1 year ago
- Between 1 year and 2 years
- Between 2 and 3 years
- 3 years or more ago
- Did not respond

SM09: Was your last mammogram performed through the Unified Health System (SUS)?

- No
- Yes
- Don't remember / Don't know
- Did not respond

Men's Health

SH01: When was the last time you had a preventive exam for prostate cancer?

- Less than 1 year ago
- From 1 year to less than 2 years ago
- 2 years to less than 3 years ago 3 years or
- more ago
- Never did
- Did not respond

SH02: What is the main reason why you have never had a prostate exam?

- ◯ I don't think it's necessary
- ◯ Shame on you
- ◯ He was never instructed to take the exam
- ◯ Don't know who to look for or where to go
- ◯ Have financial difficulties paying for transportation to the service
- ◯ Having difficulty getting transportation
- ◯ Had difficulty making an appointment
- ◯ The waiting time in the health service is very long
- ◯ The health service is very far away
- ◯ The service's operating hours are incompatible with work/domestic activities
- ◯ The health plan does not cover the exam
- ◯ It is scheduled but has not yet been carried out
- ◯ Other
- ◯ Did not respond

Specify: ______________________________

SH03: [name], was your last prostate cancer screening done through the Unified Health System (SUS)?

- ◯ No
- ◯ Yes
- ◯ Don't remember / Don't know
- ◯ Did not respond

Oral Health

SB01: When was the last time you saw a dentist?

- ◯ In the last 12 months
- ◯ From 1 year to less than 2 years
- ◯ From 2 years to less than 3 years
- ◯ 3 years or more
- ◯ Never been to the dentist
- ◯ Did not respond

SB02: Where was your last dentist appointment?

- ◯ Public service
- ◯ Private service
- ◯ Health plan or agreement
- ◯ Others
- ◯ Don't know, don't remember
- ◯ Did not respond

Specify ______________________________

SB03:What was the main reason you last saw a dentist?	◯ Never been to the dentist<br>◯ Cleaning, prevention or check-up<br>◯ Toothache<br>◯ Extraction<br>◯ Dental treatment (filling, root canal, etc.)<br>◯ Gum problem Mouth sore<br>◯ treatment Dental implant Placement/<br>◯ maintenance of<br>◯ prosthesis or dentures Other No answer<br>◯<br>◯
Specify	__________________________
SB04: Why hasn't [name] been to the dentist in the last twelve months?	◯ No need<br>◯ I had no money<br>◯ The service location was far away or there was difficulty in transportation<br>◯ There was no one to accompany him<br>◯ The health service does not have a dentist or does not operate regularly<br>◯ Incompatible schedule<br>◯ The service is very slow<br>◯ The establishment did not have a specialist compatible with its needs<br>◯ He thought he had no right<br>◯ Doesn't like the establishment's professionals<br>◯ Strike in health services<br>◯ Do you think the service does not solve your problems?<br>◯ The service was not working<br>◯ Don't know who to look for or where to go<br>◯ Other:<br>◯ Did not respond
Specify:	__________________________
SB05: In the last 6 (six) months, has [name] had a toothache?	◯ No<br>◯ Yes<br>◯ Did not respond
SB06: Regarding your teeth/mouth, [name] is:	◯ Very satisfied<br>◯ Satisfied<br>◯ Neither satisfied nor dissatisfied<br>◯ Dissatisfied<br>◯ Very Dissatisfied<br>◯ Don't know / Didn't answer<br>(Read the options)
SB07: Remembering your upper teeth, is [name] missing any teeth?	◯ No<br>◯ Yes, I lost some teeth.<br>◯ Yes, I lost all my teeth<br>◯ Did not respond
How many?	__________________________<br>(Total teeth: 16)

SB08: Remembering your lower teeth, has [name] lost any teeth?

- ◯ No
- ◯ Yes, I lost some teeth.
- ◯ Yes, I lost all my teeth
- ◯ Did not respond

How many?

____
(Total teeth: 16)

Do you use any type of dental prosthesis?

	Yes	No	Did not respond
At the top	◯	◯	◯
At the bottom	◯	◯	◯

How long have you had the upper denture?
current?

____

How long has [name] had his current lower denture?

____
(bottom)

Use of Health Services

US01: Your address is registered with the Unit of Family Health

- ◯ No
- ◯ Yes
- ◯ I don't know
- ◯ Did not respond

US02: In the last twelve months, how often has your home received a visit from a Community Health Agent or any other member of the Family Health Team?

- ◯ Monthly
- ◯ Every 2 months
- ◯ From 2 to 4 months
- ◯ Once
- ◯ Never received a visit
- ◯ Don't remember
- ◯ Did not respond

US03: In the last twelve months, how often has your household received a visit from an endemic disease agent (dengue fever, for example)?

- ◯ Monthly
- ◯ Every 2 months
- ◯ 2 to 4 times
- ◯ Once
- ◯ Never received a visit
- ◯ Don't remember
- ◯ Did not respond

US04: If [name] could, would you change teams or healthcare units?

- ◯ No
- ◯ Yes
- ◯ I would like to but there is no way
- ◯ I don't know
- ◯ Did not respond

US05: Why would you change teams or healthcare units?

- It's difficult to get to the unit
- The unit is only available/operates a few days a month
- The unit's opening hours and days do not meet the needs
- Because you can't get service You are
- poorly served
- Meet better healthcare professionals in other units
- Other units have tests that are not available in the unit where you are treated
- Others
- Did not respond

Specify: ____________________

US06: Do you have any health insurance (medical or dental), private, corporate or public?

- No
- Yes, medical AND dental coverage
- Yes, only with medical coverage
- Yes, only with dental coverage
- Did not respond

US07: How long, without interruption, have you had this health plan?

- Up to 6 months
- More than 6 months up to 1 year
- More than 1 year up to 2 years
- More than 2 years
- I don't know
- Did not respond

US08: Who pays your health plan monthly fees?

- Only the employer
- Resident of the home pays part of the plan
- Resident of the household for the entire plan
- Other family member (not living in the household)
- The interviewee himself
- Other
- Did not respond

Specify ____________________

US09: When did [name] last see a doctor?

- In the last 2 weeks
- Up to 3 months
- Up to 6 months
- Up to 1 year
- More than 1 year to 2 years
- More than 2 years to 3 years
- More than 3 years
- Never been to the doctor
- Did not respond

US10: Why has [name] not sought medical care in the past 12 months?

- ◯ No need
- ◯ I had no money
- ◯ The service location was far away or there was difficulty in transportation
- ◯ There was no one to accompany him
- ◯ Incompatible schedule
- ◯ The service is very slow
- ◯ The establishment did not have a specialist compatible with its needs
- ◯ He thought he had no right
- ◯ Doesn't like the establishment's professionals
- ◯ Strike in health services
- ◯ Do you think the service does not solve your problems?
- ◯ The service was not working
- ◯ Other
- ◯ Did not respond

Specify

____

US11: How many times have you seen a doctor in the last twelve months?

____
(999 If you don't know/don't remember/didn't answer)

US12: In which health service was your last medical consultation carried out?

- ◯ Basic Health Unit (health post or center)
- ◯ Basic River Unit (Semsa boat)
- ◯ Specialty Center, Public Polyclinic or PAM
- ◯ Emergency room or public hospital emergency room
- ◯ Private practice or private clinic
- ◯ Outpatient clinic or company/union office
- ◯ Private hospital emergency room or emergency room
- ◯ At home, with a private doctor
- ◯ At home, with a professional from the family health team
- ◯ Other service
- ◯ Don't remember
- ◯ Did not respond

US12 Specify:

____

US13: Where is this service where [name] was last seen?

- ◯ In the community itself
- ◯ In another community
- ◯ At the headquarters of Itacoatiara
- ◯ In Manaus
- ◯ Another place
- ◯ Don't remember
- ◯ Did not respond

US14: Whose means of transportation did you use the last time you went to the doctor?

- ◯ From the health service
- ◯ From another public institution, specify:
- ◯ Own
- ◯ From relatives/friends
- ◯ From other people
- ◯ From community organization/association
- ◯ Regular paid transport
- ◯ He was walking
- ◯ Other:
- ◯ Don't remember
- ◯ Did not respond

Specify

____

US15: The last time you went to the doctor in the last twelve months, how long did it take for you to get transportation?

- ◯ A day or more
- ◯ Less than 1 day
- ◯ Other:
- ◯ Did not respond

Specify:

____

US16: In the past two weeks, has [name] stopped doing any of his/her usual activities (work, school, household chores, etc.) due to health reasons?

- ◯ Yes
- ◯ No
- ◯ Did not respond

US17: For how many days?

____

US18: What was the main health reason that prevented you from carrying out your usual activities in the last two weeks?

- ◯ Back or neck pain
- ◯ Pain in the arms or hands or legs or feet
- ◯ Arthritis or rheumatism
- ◯ DORT - musculoskeletal disease related
- ◯ Headache or migraine
- ◯ Dental problem / toothache
- ◯ Cold/flu
- ◯ Asthma/bronchitis/pneumonia
- ◯ Diarrhea/vomiting/nausea/gastritis
- ◯ Dengue
- ◯ Malaria
- ◯ High blood pressure or other heart disease (heart attack, angina, heart failure)
- ◯ Diabetes
- ◯ Stroke or Stroke
- ◯ Cancer (including radiotherapy/chemotherapy)
- ◯ Depression or other mental health problem
- ◯ Injuries caused by an accident at work
- ◯ Injury caused by another type of accident
- ◯ Injury caused by assault or other violence
- ◯ Other health problem:
- ◯ Did not respond

Specify:

____

US19: Have you been bedridden in the past two weeks?

- ◯ Yes
- ◯ No

US20: How many days were you bedridden?	______________________________
US21: Do you usually go to the same place, the same doctor or the same health service when you need health care?	◯ Yes<br>◯ No
US22: When [name] is sick or in need of health care, where do they usually go first?	◯ Pharmacy or Drugstore<br>◯ Basic Health Unit (health post or center)<br>◯ Basic River Unit (Semsa boat)<br>◯ Specialty Center, Public Polyclinic or PAM<br>◯ Emergency room or public hospital emergency room<br>◯ Private practice or private clinic<br>◯ Outpatient clinic or company/union office<br>◯ Hospital emergency room or emergency room private<br>◯ At home, with a private doctor<br>◯ At home, with a healthcare professional of the family<br>◯ Other service:<br>◯ Did not respond
Specify:	______________________________
US23: Where is this service that you usually look for?	◯ In the community itself<br>◯ In another community<br>◯ At the headquarters of Itacoatiara<br>◯ In Manaus<br>◯ Other:<br>◯ Did not respond
Specify	____________________________________
US24: How long does it take to get from your home to the service you usually look for?	◯ A day or more<br>◯ Less than a day, on average ______ minutes<br>◯ Don't know/don't remember<br>◯ Did not respond
Specify how many minutes	______________________________<br>(3h = 180min; 5h =300min; 8h = 480min)
US25: How do you get to the service you usually seek?	◯ On foot<br>◯ By boat/tailboat/speedboat<br>◯ Bicycle<br>◯ Own motorcycle<br>◯ Own car<br>◯ Taxi<br>◯ Health service vehicle<br>◯ Vehicle of another public institution (specify institution)<br>◯ Vehicle of relatives/friends<br>◯ Community organization/association vehicle<br>◯ Other:<br>◯ Did not respond

Specify:

____

US27: Do you consider the distance from your home to this health unit:

- ◯ Far away, but easy to access
- ◯ Far away and difficult to access
- ◯ Close, easy access
- ◯ Close, but difficult to access
- ◯ Did not respond

(Read the options)

[us22]

____

US28: How long ago was your last appointment?
in that unit that [name] usually looks for?

____

US29: How did you get your last appointment at this unit?

- ◯ Return, scheduled at the previous appointment
- ◯ Through the community agent
- ◯ Through another health professional
- ◯ He went on his own and waited on the day to be attended
- ◯ Other:
- ◯ Did not respond

Specify

____

US30: How do you rate the level of difficulty in obtaining care at this health unit:

- ◯ Very easy
- ◯ Easy
- ◯ Neither easy nor difficult
- ◯ Difficult
- ◯ Very difficult
- ◯ Did not respond

(Read the options)

US31: How long did you wait on the day of the
last appointment, to be seen?

____
(Write down in minutes. 2h=120; 3h=180; 5h=300)

US32: How would you rate the care you received from the healthcare professional during your last consultation:

- ◯ Very good
- ◯ Good
- ◯ Regular
- ◯ Bad
- ◯ Very Bad
- ◯ Doesn't remember / Doesn't know / Didn't answer

US33: Have you ever been referred by professionals at
this unit for other services (e.g. specialist, exams)?

- ◯ No
- ◯ Yes
- ◯ Don't remember
- ◯ Did not respond

US34: When you needed to be referred to other services, how was the appointment scheduled?

- ◯ By the health unit and informed at the time
- ◯ By the health unit and informed later
- ◯ Scheduled by the health department and informed later
- ◯ You must call or go to the specialist appointment scheduling center
- ◯ Receive a referral form and seek the service on your own
- ◯ Don't remember / Don't know
- ◯ Did not respond

US35: In the last 12 months, were you admitted to a hospital for 24 hours (a whole day) or more?

- ◯ Yes
- ◯ No

US36: How many times has [name] been hospitalized?

____
(In the last 12 months)

US37: What was the reason for your last hospitalization?

- ◯ Clinical treatment
- ◯ COVID
- ◯ Psychiatric treatment
- ◯ Surgery
- ◯ Complementary diagnostic tests
- ◯ Others:
- ◯ Did not respond

Specify

____

US38: How long were you hospitalized?

____
(In days)

[us22]

____

US39: If you are unable to get care at the location you usually go to, what is the second option you usually look for?

- ◯ Pharmacy or Drugstore
- ◯ Basic Health Unit (health post or center)
- ◯ Basic River Unit (SEMSA boat)
- ◯ Specialty Center, Public Polyclinic or PAM
- ◯ Public hospital emergency room or emergency room
- ◯ Public hospital/outpatient clinic
- ◯ Private practice or private clinic
- ◯ Outpatient clinic or company/union office
- ◯ Private hospital emergency room or emergency room
- ◯ At home, with a private doctor
- ◯ At home, with a professional from the family health team
- ◯ Other service:
- ◯ Nowhere
- ◯ Did not respond

Specify

____

US40: Where is this service that you usually look for as a second option?

- ☐ In the community itself
- ☐ In another community
- ☐ At the headquarters of Itacoatiara
- ☐ In Manaus
- ☐ Other:
- ☐ Did not respond

Specify: ____________________

US41: Before seeking any health service, do you usually do anything on your own, at home or in the community to solve your problem or reduce your suffering?

- ○ Yes
- ○ No
- ○ Did not respond

US42: Which of these treatments do you usually use:

	Yes	No	Did not respond
Pharmacy medicine that already had at home	○	○	○
Homemade medicine (grown at home or in the community)	○	○	○
Medicine that you searched the forest and prepared	○	○	○
Medicine made by a healer of community	○	○	○
Prayer woman/blesser	○	○	○
Others:	○	○	○

Specify ____________________

US43: Even though you are receiving healthcare services, do you usually use these resources (home remedies, prayers) along with medical treatment?

- ○ No
- ○ Yes
- ○ Sometimes
- ○ Did not respond

US44: Where do you usually have your laboratory tests performed?

- ○ In the health unit that serves the community
- ○ Elsewhere for free:
- ○ Pay for the exam
- ○ Don't know/don't remember
- ○ Never had lab tests
- ○ Did not respond

Specify ____________________

[name], I'm going to ask you some questions about household items for economic classification purposes.
All the items I will mention must be working, including those that are stored.

NUMBER OF ASSETS

	0	1	2	3	4	5	6	7	8+	IGN
RB1: Color Television:	○	○	○	○	○	○	○	○	○	○
RB2: Refrigerator:	○	○	○	○	○	○	○	○	○	○
RB3: Washing machine:	○	○	○	○	○	○	○	○	○	○
RB4: Landline/conventional telephone:	○	○	○	○	○	○	○	○	○	○
RB5: Cell phone:	○	○	○	○	○	○	○	○	○	○
RB6: Computer (including notebook):	○	○	○	○	○	○	○	○	○	○
RB7. Motorcycle:	○	○	○	○	○	○	○	○	○	○
RB8: Car:	○	○	○	○	○	○	○	○	○	○
RB9: Microwave oven:	○	○	○	○	○	○	○	○	○	○
RB10: DVD player: RB11:	○	○	○	○	○	○	○	○	○	○
Freestanding freezer: RB12:	○	○	○	○	○	○	○	○	○	○
Dishwasher RB13. Clothes	○	○	○	○	○	○	○	○	○	○
dryer	○	○	○	○	○	○	○	○	○	○
V42. Air conditioning:	○	○	○	○	○	○	○	○	○	○
V40. Fan:	○	○	○	○	○	○	○	○	○	○
V38. Bicycle:	○	○	○	○	○	○	○	○	○	○
V17. AM/FM Radio:	○	○	○	○	○	○	○	○	○	○
V18. Sound system/speaker: V37.	○	○	○	○	○	○	○	○	○	○
Satellite dish: V22. Gas	○	○	○	○	○	○	○	○	○	○
stove: V35. Power	○	○	○	○	○	○	○	○	○	○
generator:	○	○	○	○	○	○	○	○	○	○
V36. Solar panel:	○	○	○	○	○	○	○	○	○	○
V27. Chainsaw:	○	○	○	○	○	○	○	○	○	○
V41. Brush cutter:	○	○	○	○	○	○	○	○	○	○
V29. Pack/work animal (horse/donkey/ ass/ox):	○	○	○	○	○	○	○	○	○	○
V30. Outboard engine:	○	○	○	○	○	○	○	○	○	○
V31. Tail:	○	○	○	○	○	○	○	○	○	○
V32. Canoe:	○	○	○	○	○	○	○	○	○	○
V33. Aluminum boat:	○	○	○	○	○	○	○	○	○	○
V39. Cassava grater with motor:	○	○	○	○	○	○	○	○	○	○

Relevant observations

____
